# A meta-analysis of the association between physical demands of domestic labor and back pain among women

**DOI:** 10.1186/s12905-021-01294-5

**Published:** 2021-04-13

**Authors:** Abisola Osinuga, Chelsea Hicks, Segun E. Ibitoye, Marin Schweizer, Nathan B. Fethke, Kelly K. Baker

**Affiliations:** 1 Department of Occupational and Environmental Health, University of Iowa, Iowa City, 52242 USA; 2 Department of Health Promotion and Education, University of Ibadan, Ibadan, 200212 Nigeria; 3 Department of Epidemiology, University of Iowa, Iowa City, 52242 USA

**Keywords:** Domestic labor, Women, Physical demands, Biomechanical, Back pain, Meta-analysis

## Abstract

**Background:**

Back pain (BP) is among the most common musculoskeletal problems globally and is a leading contributor to disability among adults. Millions of women especially those in low-income settings, engage in strenuous domestic activities that may increase their risk of BP. The purpose of this meta-analysis was to estimate the association between physically demanding domestic labor (PDDL) which is characterized as intensity, frequency, duration of work and biomechanical risk factors of work and BP among women.

**Methods:**

Five databases were searched for records published from January 1991 to March 2020; and results from 11studies were included in the meta-analysis. A random effects model and the generic inverse-variance method was used to estimate the pooled odds ratio (OR), 95% confidence interval (CI), and the degree of heterogeneity among studies (I^2^). Stratified and sensitivity analyses were conducted to identify the influence of outliers and identify the sources of heterogeneity.

**Results:**

Exposure to high PDDL was significantly associated with BP (OR = 1.63; 95% CI 1.30, 2.04; I^2^ = 70%). The odds of back pain were highest among the following groups: women performing domestic labor in non-neutral postures (OR = 2.30; 95% CI = 1.75–3.04; I^2^ = 0%; N = 4 studies) and among women from low- and middle-income countries (OR = 1.98; 95% CI = 1.58–2.49; I^2^ = 29%; N = 5 studies). We found no evidence of publication bias (Egger’s test p-value = 0.15).

**Conclusions:**

PDDL may significantly increase a woman’s risk of experiencing BP, but larger prospective studies are needed to further investigate the association. Presenting data on how domestic work affects the musculoskeletal health of women will be important in designing future interventions (behavioral, infrastructural, and ergonomic) that can reduce the burdens from domestic labor.

**Supplementary Information:**

The online version contains supplementary material available at 10.1186/s12905-021-01294-5.

## Background

Back pain (BP) includes acute (less than 6 weeks), chronic (pain lasting more than 12 weeks), or neuropathic pain in the upper or lower back. BP is a leading contributor to disability and activity limitation and the main contributor to the overall burden of musculoskeletal disorders and injuries (MSDs) [1]. In addition, low back pain (LBP) was ranked as the highest contributor of years lived with disability (YLDs) among adults globally, accounting for 64.9 million YLDs in 2017 [2]. Although BP can be a self-limiting condition, the re-occurrence rate is high (approximately 60%) and individuals are likely to experience another episode within 3–6 months [2, 3]. Limitation of activities because of LBP may result in loss of productive work time, increased medical expenditure, and further entrenchment of low-income individuals in poverty [4, 5].

The most common risk factors for BP include demographic characteristics, personal health habits, psychological factors, occupational exposures, and other chronic comorbidities [4, 6–11]. Prior reviews have established heavy manual work, non-neutral work postures (bending and twisting, repetitive motions, and long working hours) as risk factors for BP among occupational populations [8, 9, 12, 13]. Biological sex and gender are also risk factors; females have a higher prevalence of LBP in the general population than men [14–17]. A global burden of disease study found that the age-standardized prevalence of LBP is also higher among women than men [2].

The gendered difference in BP has been attributed to several occupational factors, such as differential exposures to work-related physical and physiological factors, male-oriented tool and workstation designs, and gendered variation in the perception of pain [11, 18–20]. Generally, women tend to be clustered in specific occupations with different pattern of employment and exposures from those of men [21]. However, several occupational studies and reviews have shown that women-dominated jobs may be just as physically taxing as male-dominated jobs [11, 16, 17, 22–24]. Thus, women’s unique occupational exposures may place them at risk for MSDs, including BP [21].

Additionally, millions of women experience strenuous daily work conditions in domestic labor, often in addition to a formal job. Women’s domestic labor, which involves tasks such as cleaning, cooking, water fetching, manual washing of clothes, and family care duties, may be as physically, emotionally and time demanding as structured paid work [25–29]. In some low- and middle-income countries (LMICs), women spend an average of 10 h per day engaged in strenuous domestic task in awkward postures [30]. Yet, few studies have examined the effects of the physical demands of domestic labor (PDDL) on the musculoskeletal health of women. Social norms dictate domestic work as a woman’s duty in many LMICs. This may discourage spousal and familial involvement in domestic roles even while women increasingly participate in the paid workforce. The double burden of exposure from paid work and domestic labor is particularly problematic among low-income populations where there are limited social services to relieve burdens [28, 31, 32].

Few systematic reviews have assessed gender-specific relationships between PDDL and BP in the general population [33, 34]. Previous meta-analyses of the relationship between non-occupational physical activities (such as sporting, commuting and domestic physical activities) and BP combined results from different types of non-occupational physical activities and did not present stratified results from domestic labor [33, 35]. Some of these reviews were also limited to studies published in English [33, 36].

Currently, no meta-analysis has assessed the association between PDDL and BP among women. This systematic review aims to fill this gap by examining the contribution of PDDL to the risk of BP among women. We included research studies that both addressed exposures from domestic labor exposures and presented female-specific effect estimates. We also conducted subgroup analysis by country type (high income countries versus LMICs) and definitions of exposure to explore how these issues influence the relationship between PDDL and BP.

## Methods

### Design and search strategy

A systematic literature search was conducted to identify records published from January 1991 to March 2020 in PubMed, Embase, Web of Science, Scopus, and CINAHL, using search terms and keywords related to the population of interest, the exposure, and the outcome, without language restrictions (see “Appendix in online supplemental files”). Google Scholar and the reference lists of relevant articles were searched for additional citations. This meta-analysis was performed and reported based on the Meta-analyses of Observational Studies in Epidemiology (MOOSE) criteria and in accordance with the Preferred Reporting Items for Systematic Reviews and Meta-Analyses (PRISMA) checklist [37].

### Eligibility criteria

#### Inclusion

Included studies were those that were published in peer-reviewed journals; assessed exposures from non-occupational domestic labor; focused specifically on women, or women represented at least 50% of study sample and presented gender-stratified effects; described domestic tasks done by women; and reported data needed to derive the measure of effect and the corresponding 95% CI.

#### Exclusion

Excluded studies were those that were anecdotal, case series, editorials, and reviews; focused only on occupational domestic work; included only elderly (over age 65 years) or pregnant women; included acute musculoskeletal injuries (e.g., from slips/trips/falls or other traumatic events) or focused on MSDs in other body areas (e.g., shoulder pain and carpal tunnel syndrome) as the outcome; included participants with BP secondary to a specific disease (e.g., osteoporosis and cancer); described exposure based solely on the woman’s role (e.g., “housewives”) but not on PDDL; focused on caregiving for disabled persons; or did not report effect estimates separately for non-occupational domestic labor.

### Data extraction

Two reviewers (AO and CH) independently reviewed and abstracted information from potential studies after duplicate reports had been removed. Disagreements were resolved by consensus and discussion with a third reviewer (SI). The following information was abstracted: author, the year of publication, country of study, study design, proportion of the sample that included women, results stratified by gender (yes/no/not applicable), at least one type of domestic tasks is specified (yes/no), mean age of women participants, study sample size, numbers in exposed and unexposed groups, definition of BP, measures of domestic work exposures. One study that was published in Italian [52], was translated to English using Google Translate.

### Assessment of study quality

The Newcastle–Ottawa Scale for Observational Studies [38] was used to evaluate the risk of bias among the studies identified for inclusion. Some modifications were made to the scale based on the study design (Additional file 1: Table S1). For example, criteria for exposure definition were modified in the scale. The assessment was conducted by two reviewers (AO and CH) independently. Disagreements were resolved by discussion or consultation with a third reviewer (SI). The scale, totaling 10 points, evaluates the risk of bias from three domains: the selection of the study sample (representativeness of sample/cases, adequacy of sample size, response rates, definition and ascertainment of exposure and definition of cases and controls), comparability of study groups (potential confounding variables), and conceptualization of outcome (outcome definition and ascertainment, appropriateness of statistical measures). Quality scores were categorized as high (≥ 8 points), moderate (6–7 points), or low (≤ 5 points). For the risk of bias plots, the Robvis visualization tool was used to categorize and color-code each element in the three domains based on high risk, some concerns, and low risk of bias [39].

### Definition of outcome and exposure variables

The outcome was defined as either self-reported pain/discomfort in the back area (upper, lower, or non-specific) or care-seeking for BP. We included studies regardless of recall period used to ascertain self-reported back pain status. PDDL, which is the exposure variable, were expected to be assessed differently across studies. Work exposures are typically characterized in terms of frequency, intensity, and duration, and studies rarely assess the three dimensions of exposure at once [40]. Therefore the exposure variable included those that categorized PDDL in terms of the intensity of labor (heavy or light PDDL), or assessed the duration of domestic labor (time spent on domestic labor per week/per day), or assessed the frequency/time spent working in awkward postures or history of forceful lifting.

### Data synthesis and statistical analysis

The primary analytic objective was to estimate, using exposure and outcome data abstracted from each included study, a pooled estimate of association PDDL and BP. We also conducted subgroup analyses by country status (high versus low- and middle-income countries), by definitions of exposure (time, frequency/intensity of labor, and biomechanical factors), and focus on gender (women only versus studies with both male and female included). If a study examined all the types of PDDL exposure, only the biomechanical exposure was analyzed. Stratified analyses were performed based on the study quality (i.e., high/moderate/low) and whether effect estimates were adjusted to control for confounding (yes/no).

Since most studies used a cross-sectional design, the OR was used as the measure of association. Measures of effect from included studies were pooled using the natural logarithm of the ORs (logOR), and the OR from each study was weighted by the inverse of its variance. Unadjusted ORs were used if adjusted ORs were not provided. When no effect estimate was given, the unadjusted OR was calculated directly from the abstracted information. A random-effects model was used to estimate the pooled OR and the 95% CI [41]. The data were pooled in Microsoft Excel, analyzed in the Review Manager (RevMan) version 5.3 program [42] and with the package ‘meta’ in R [43].

Heterogeneity among studies was examined by using the Cochran’s Q test and quantified using the Higgins I^2^ statistic [44]. We set the criterion for a statistically significant Cochrane Q test to *p* < 0.1. The degree of heterogeneity was defined as low (I^2^ <  = 25%), moderate (26–50%), high (I^2^ = 51–75%), and very high (I^2^ > 75%). Publication bias was assessed using funnel plots and Egger’s test [45, 46]. The Leave-one-out method, and Baujat Plot, were used to investigate the effect of outliers and influencers on the degree of heterogeneity. We conducted sensitivity analyses to examine the influence of outliers and influencers, studies with low quality scores, or those that did not adjust for confounders on the Pooled OR.

## Results

### Study selection process

**Fig. 1:**
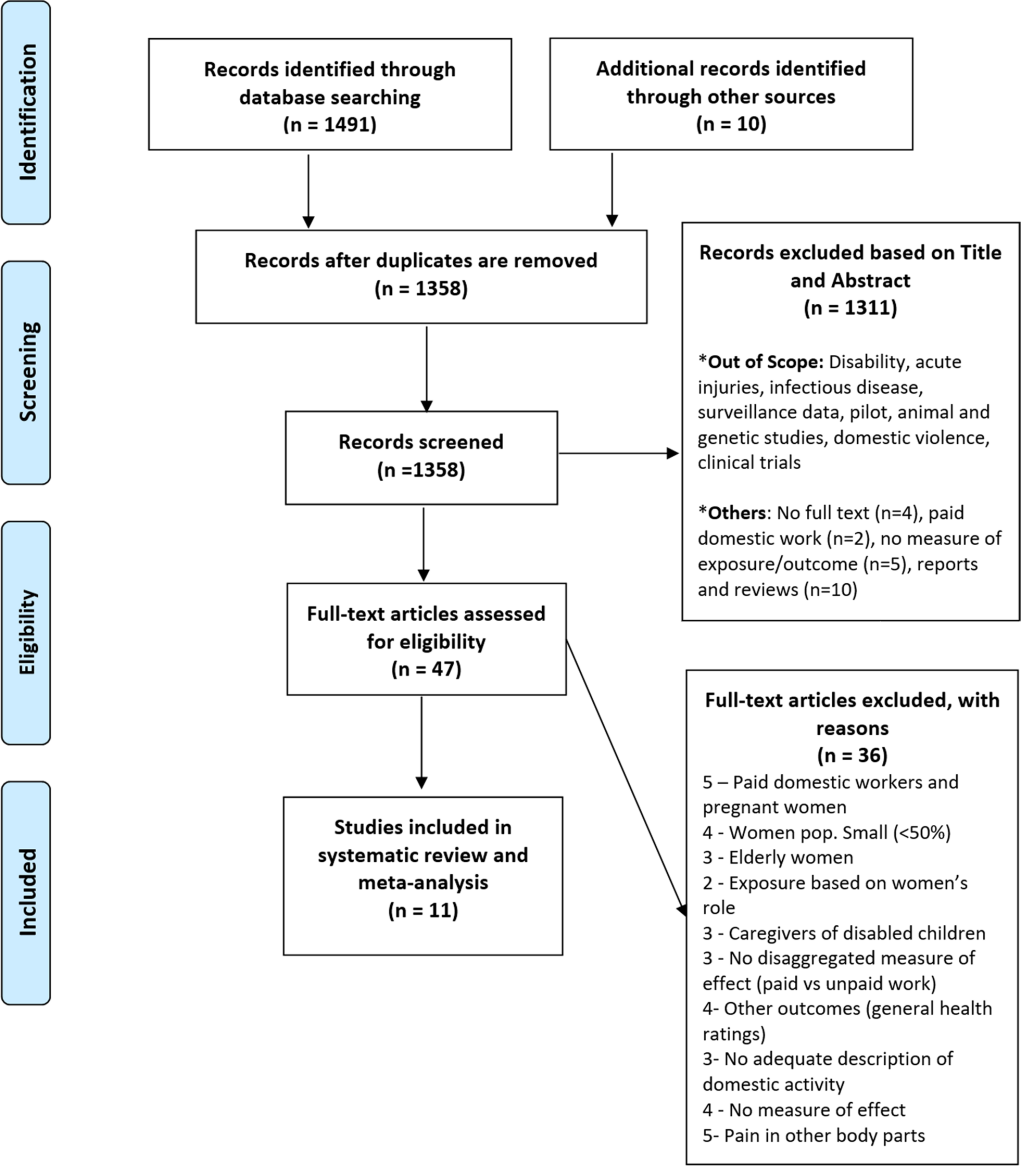
Flow of studies through the review

Figure 1 shows the detailed results of the identification and study selection process. We retrieved 1,358 non-duplicate records through search of databases and references from relevant articles. Following review of titles and abstracts 1311 studies were excluded. Forty-seven full text articles were subsequently assessed for eligibility and 36 were excluded as shown in Fig. 1 and Additional file 1: Table S4.

### Study characteristics

Additional file 1: Table S1 summarizes the characteristics of included studies. There were 101,394 total women included across the eleven studies, with sample sizes ranging from 73 to 64,348 women. Mean age of participants ranged from 29 to 49 years across studies. Six studies included samples from high-income countries [47–52] and five studies were included samples from LMICs (Ghana/South Africa, Bangladesh, Sri Lanka, Brazil, Lebanon) [25, 53–56].

Seven studies recruited only women [25, 47, 49, 51–53, 56], three studies either had a sample that included at least 50% women or reported gender-stratified results [48, 50, 54], and one large-scale study did not specify the number of women recruited but stratified results by gender [55]. All studies specified common domestic tasks done by women (e.g. cleaning, cooking, child caregiving). Four studies were on full-time housewives/homemakers [25, 49, 53, 56] while the remaining eight included women with paid employment.

Three studies categorized PDDL as number of hours per week performing domestic tasks [49–51] four studies categorized exposure as biomechanical (lifting, carrying, working in static and awkward postures) [25, 53, 54, 56] and the rest categorized PDDW based on intensity (heavy or light) and frequency of labor [47, 48, 52, 55]. In all included studies, exposure information was ascertained by self-report (i.e. questionnaire). Eight out of eleven studies included in this review were on low back pain while the remaining three were on chronic back pain, care-seeking for low back pain and upper back pain. The only case–control study defined outcome as care-seeking for low back pain [51] while the rest used self-reported (7 days, 1, 3, or 12 months) BP. All studies except one reported ORs adjusted for relevant common confounders such as age, education, occupation, income, and psychosocial factors of paid work.

### Quality assessment

**Fig. 2:**
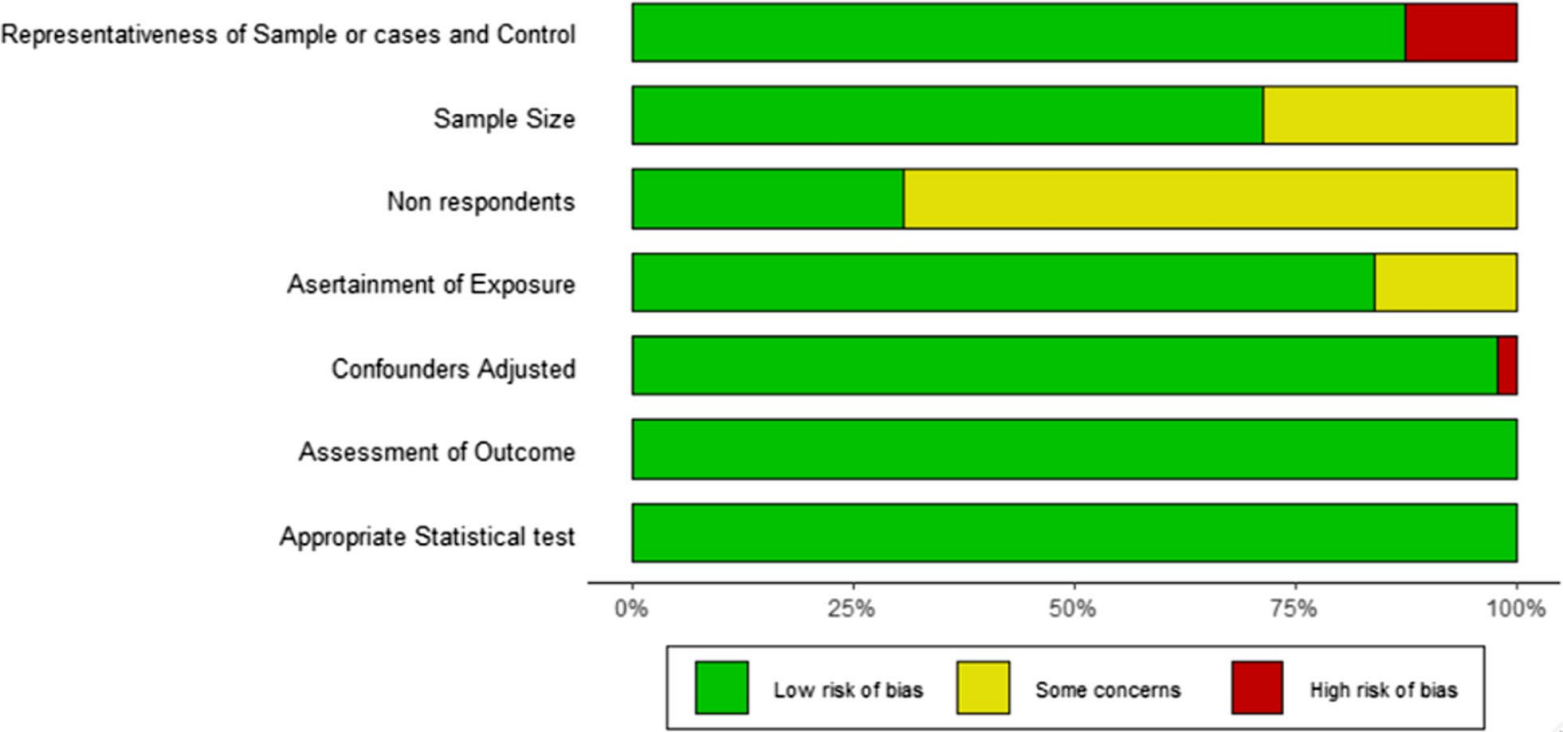
RobVis (risk of bias visualization) plot

Using the Newcastle Ottawa Scale, six studies were categorized as high quality (8 points and above), four as moderate quality (6 and 7 points), and one as low-quality (5 points). The Robvis visualization plot in Fig. 2 shows that most studies have low risk of bias in most of the sub-domains except for assessment of non-respondents (a subdomain of selection of study participants). The quality assessment table and Robvis plot are depicted in Additional file 1: Table S2 and Fig. 2, respectively.

### Overall association between PDDL and BP

**Fig. 3:**
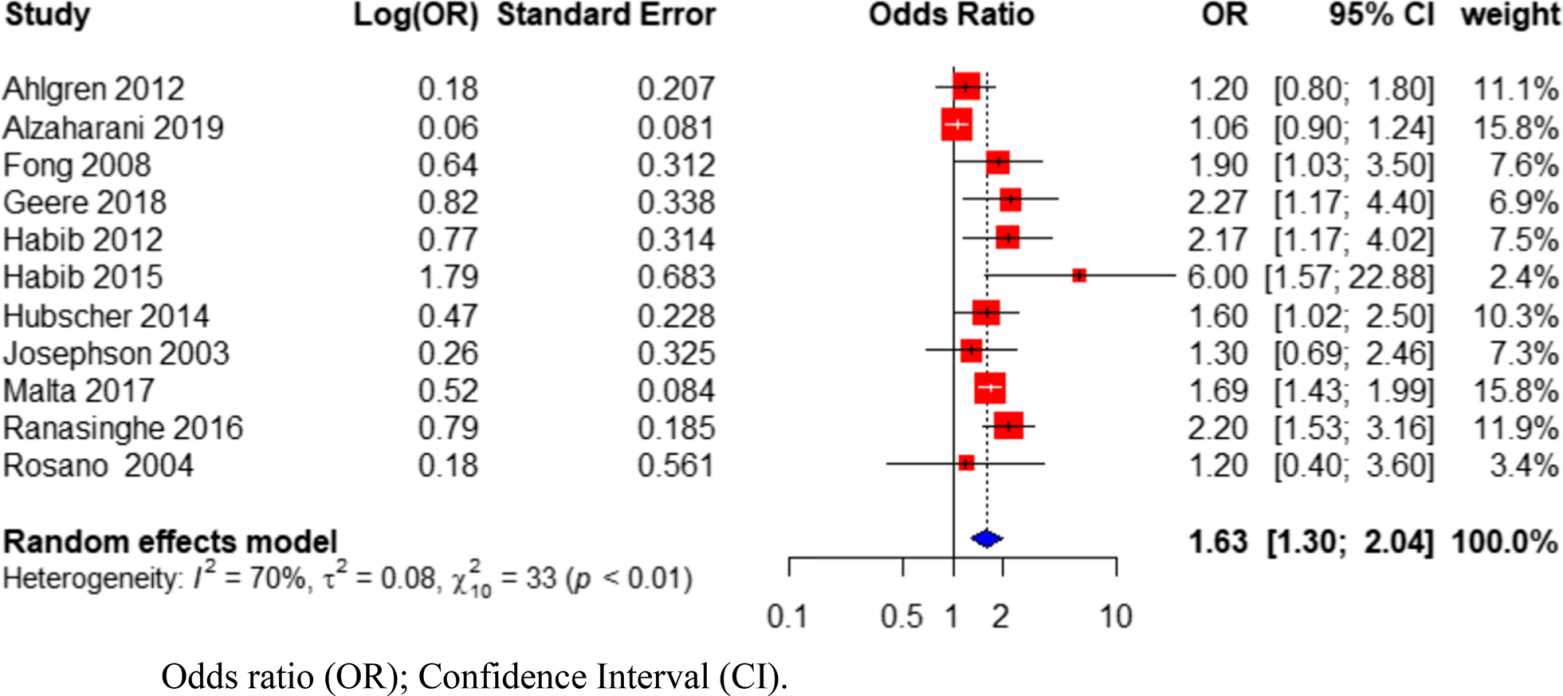
Forest plot of all studies investigating the association between physical demands of domestic labor and back pain in women

The pooled odds ratio from eleven studies showed that high PDDL, characterized as long duration, high frequency and intensity, or high biomechanical demand (awkward posture and lifting heavy objects), was significantly associated with BP among women (OR = 1.63; 95% CI = 1.30–2.04; Fig. 3). However, there was substantial heterogeneity among the included studies (Cochrane Q-test *p* value < 0.01; I^2^ = 70%; Fig. 3).

### Identifying and quantifying heterogeneity

#### Outlier and influential analysis

The Baujat diagnostic plot (Additional file 1: Figure S1) showed that Alzaharani et al. [48] contributed to the overall heterogeneity to the greatest extent and had the most influence on the overall pooled OR, likely due to the large sample size compared to the other studies. Influential analysis, using the leave-one out method (Additional file 1: Figure S2), also revealed that Alzaharani et al. [48] was the predominant source of heterogeneity. The lowest I^2^ value (16%) was observed when the Alzaharani et al. [48] was removed from the analysis. Omitting Habib and Rahman [53] the study with the greatest effect size (OR = 6.00; 95% CI = 1.57–22.88), did not meaningfully influence the pooled effect size (OR = 1.57; 95% CI = 1.26–1.95; Additional file 1: Figure S2) or the degree of heterogeneity (I^2^ = 69%).

#### Subgroup analysis

**Fig. 4:**
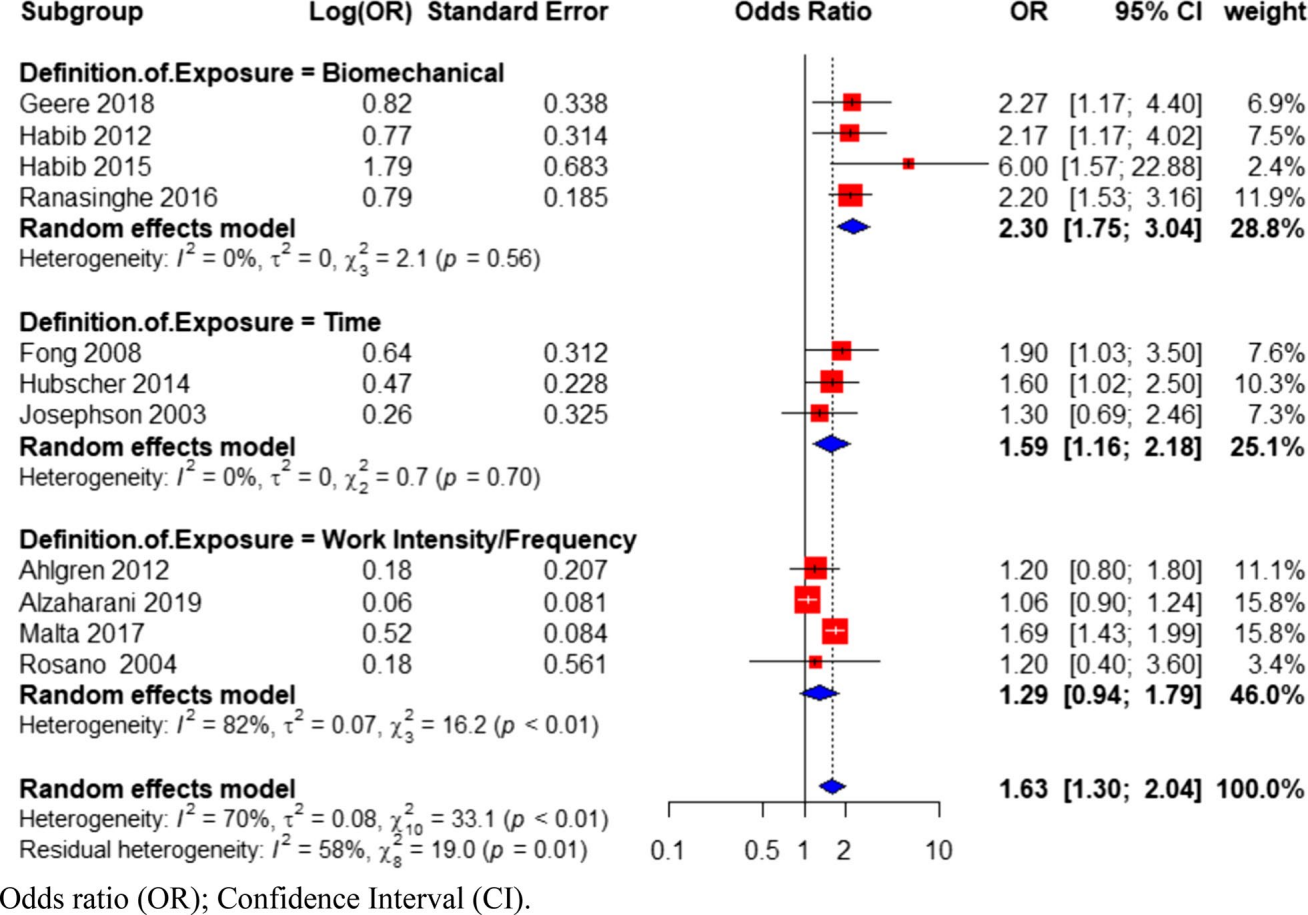
Forest plot of subgroup analysis based on exposure definition, investigating the association between physical demands of domestic labor and back pain in women

Subgrouping studies based on definitions of exposure (hours/week of domestic labor, frequency, or intensity of domestic task per week, and presence of biomechanical factors such as lifting, carrying and awkward postures), resulted in variation of the pooled odds ratio across groups (1.29–2.30) (Fig. 4). The test for between-group heterogeneity was statistically significant (Q = 7.48, *p *value = 0.02 (Additional file 1: Table S3). Within-group heterogeneity was reduced for studies that defined exposure as a function of time (OR = 1.59; 95% CI 1.16–2.18; I^2^ = 0%; N = 3 studies) or by biomechanical exposure (OR = 2.30; 95% CI 1.75–3.04; I^2^ = 0%; N = 4studies) (Fig. 4), compared to studies that defined exposure based on work intensity or frequency (OR = 1.29; 95% CI 0.94–1.79; I^2^ = 82%; N = 4 studies), due to the influential effect of Alzaharani et al. [48].

**Fig. 5:**
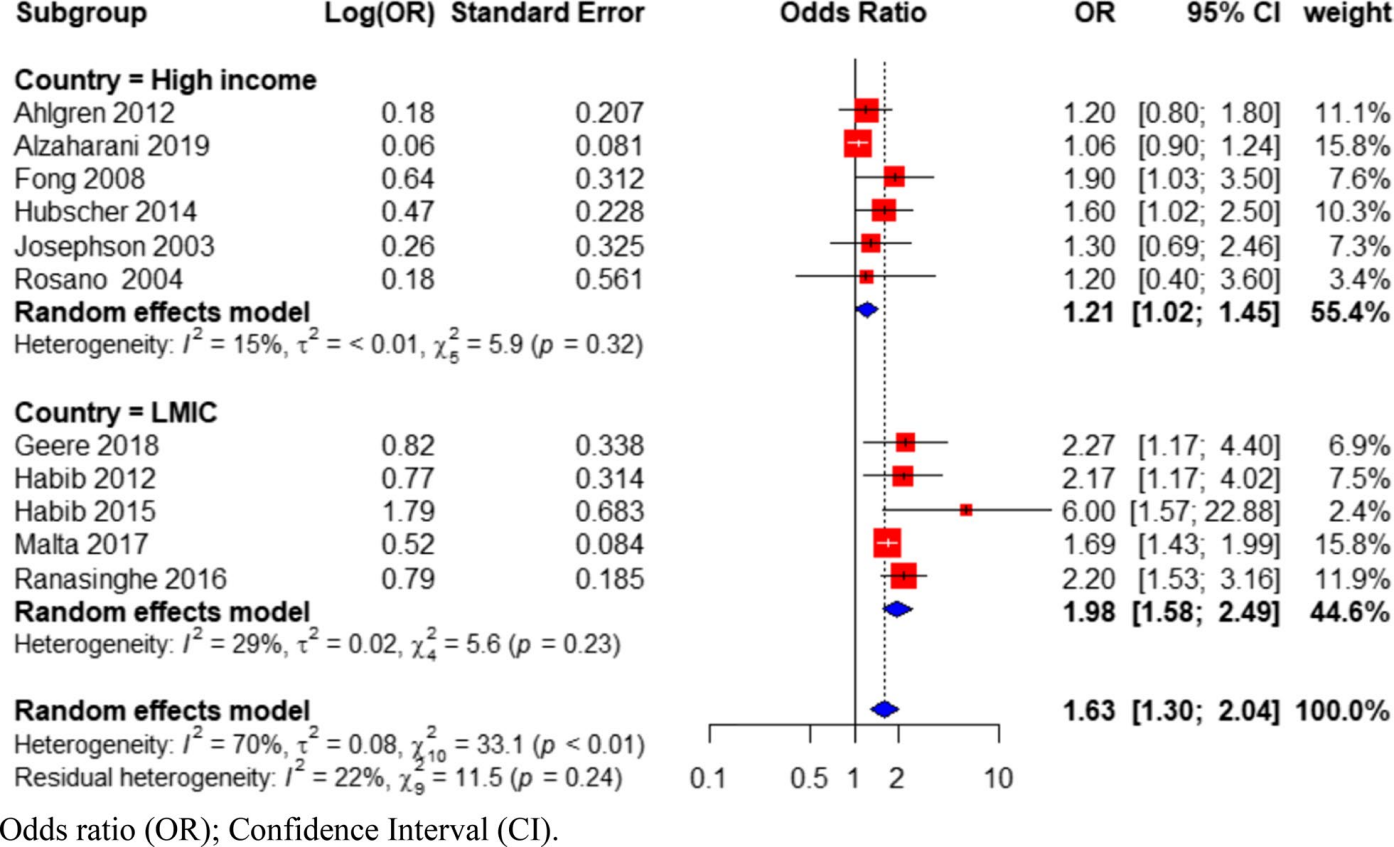
Forest plot of subgroup analysis based on country status, investigating the association between physical demands of domestic labor and back pain in women

**Fig. 6:**
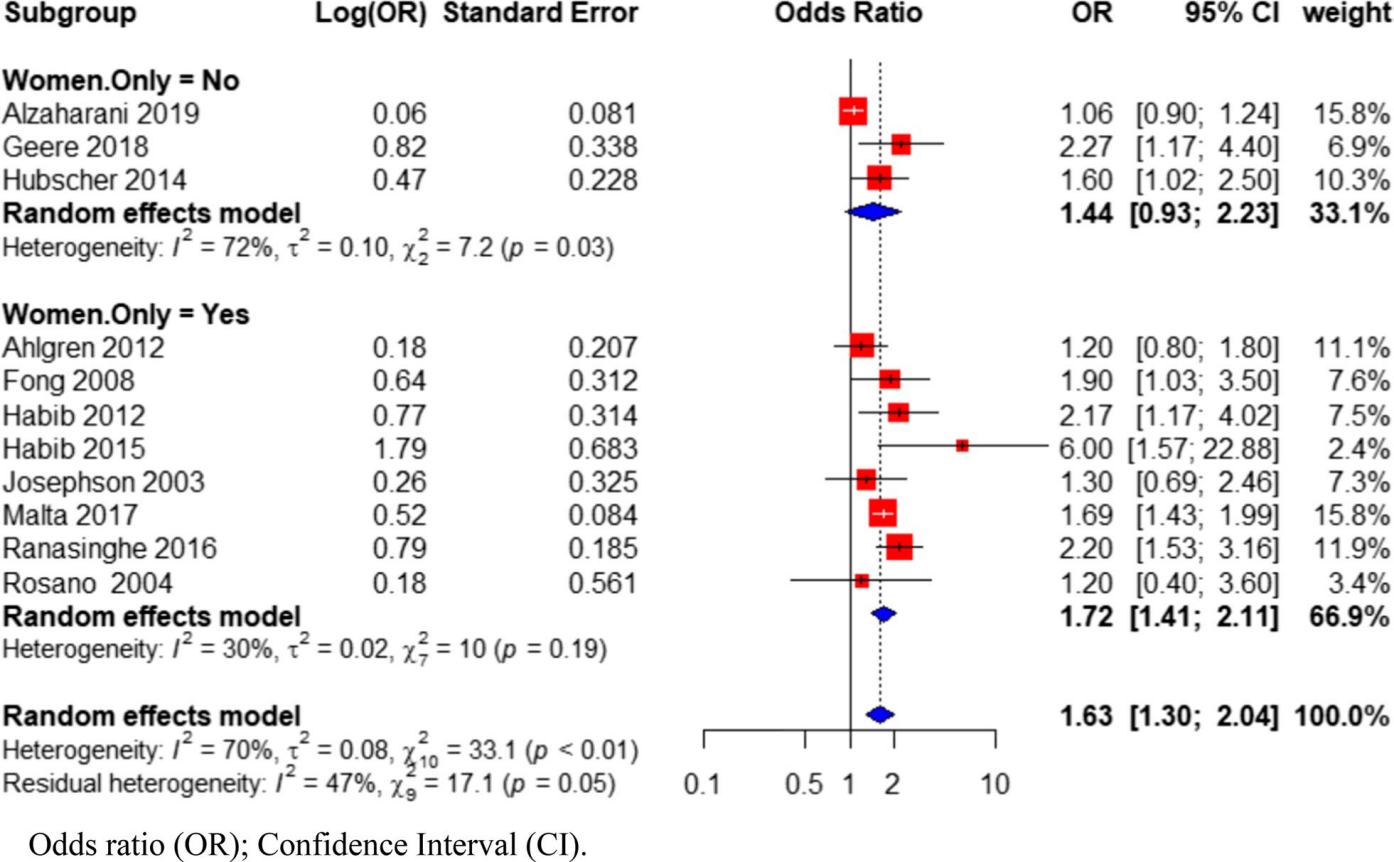
Forest plot of subgroup analysis based on study population, investigating the association between physical demands of domestic labor and back pain in women

Subgrouping studies based on country status (Fig. 5) showed that there was significant between-group heterogeneity (Q = 11.22; p = 0.0008; Additional file 1: Table S3). Within-group heterogeneity was reduced among high-income (OR = 1.21; 95% CI 1.02–1.45; I^2^ = 15%; N = 6 studies) and LMICs subgroup (OR = 1.98; 95% CI 1.58–2.49; I^2^ = 29%; N = 5 studies) when compared to the heterogeneity (I^2^ = 70%) within all included studies as shown in Fig. 5. Studies from LMICs had a stronger association between PDDL and back pain than studies from high-income countries. When studies were sub-grouped by gender specificity (Fig. 6), studies with samples of only women had a stronger association between PDDL and back pain (OR = 1.72; 95% CI 1.41–2.11; I^2^ = 30%; N = 8 studies) than studies with samples of both men and women (OR = 1.44; 95% CI 0.922.23; I^2^ = 72%; N = 3 studies).

#### Stratified analysis

Compared to the overall pooled OR of 1.63, slightly lower effect sizes were observed when considering only studies categorized as high quality (OR = 1.54 95% CI 1.18–2.02; I^2^ = 80%; N = 6 studies, Additional file 1: Figure S3) and when omitting the one study [53] (Habib MM and Rahman 2015) that did not provide an adjusted OR (OR = 1.57; 95% CI = 1.26–1.95; I2 = 69%; N = 10 studies; omitting Habib MM and Rahman (2015), in Additional file 1: Figure S2). Heterogeneity increased from 70% (I^2^ from pooled effect estimate) to 80% when the pooled analysis included only high-quality studies (Additional file 1: Figure S3). When stratifying by type of study design, excluding the only case–control study Josephson et al. [51] did not meaningfully influence pooled effect estimate nor the degree of heterogeneity (OR = 1.66,95% CI = 1.31–2.11; N = 10 studies; Additional file 1: Figure S2).

#### Publication bias

The funnel plot, a plot of the odds ratios against the standard errors from each included study appears symmetrical, suggesting limited evidence of publication bias (Additional file 1: Figure S4). This conclusion is also supported by the result of the Egger’s test (*p* = 0.15).

## Discussion

### General discussion

To the best of our knowledge, this is the only synthesis of observational studies that assessed the association between PDDL and BP while comparing estimates based on country status and standardized dimensions of physical work exposures (frequency, duration, and magnitude). Our results demonstrated that women who perform high PDDL, characterized as long-time spent laboring, frequency, and magnitude (heavy workload) and the presence of biomechanical risk factors (activity conducted in non-neutral posture) have a higher odd of BP compared to women who perform low PDDL. The magnitude and direction of the pooled estimates from the overall and subgroup analyses did not change meaningfully when the major source of heterogeneity was omitted and when analyses were restricted to high quality studies.

Standard operationalization of biomechanical exposure comprises three main dimensions; level or magnitude, repetitiveness or frequency, and duration of work. Exposures in the included studies were described in self-reported magnitudes, frequency and duration of domestic tasks such as carrying, lifting, pushing as well as self-reported frequency or duration of working in awkward postures and lifting. Biomechanical loading from manual material handling activities and working in awkward postures are established risk factors for both recurrent and chronic back pain [34]. This agrees with our results, specifically that the pooled OR from studies in which exposures were defined based on biomechanical characteristics of domestic labor was greater than the pooled ORs from studies in which exposures were defined in more generic terms (e.g., time spent performing domestic labor).

We did not identify any prospective studies to include in the analyses, and a previous review of both occupational and non-occupational risk factors for LBP did not include exposures from domestic labor [34]. We are unaware of other studies assessing the association between PDDL among women specifically, although one review of prospective studies found that women in the general population had a higher prevalence of low back pain compared to men [11]. The gender differences in prevalence of BP could be due in part to women in many regions of the world being disproportionately exposed to PDDL. The results of this study suggests that PDDL and its musculoskeletal health risks should be more recognized and examined in occupational research, especially in LMICs where millions of low-income women are exposed to strenous daily work conditions that are comparable to work in occupational environments.

A prior systematic review of 35 studies assessed the association between occupational lifting and low back pain and concluded that it is unlikely that occupational lifting is independently associated with LBP [13]. Another systematic review found no significant relationship between free-living physical activity (classified as leisure-time physical activity) and non-specific LBP [57]. The result of the former review may be different from ours because we included studies that examined non-neutral postures as biomechanical risk factors rather than just the activity of lifting. Also, our study assessed domestic physical activity among women in the household, not workplace physical activity. For the latter prior review, domestic physical activity was classified as a component of leisure time physical activity, which could have influenced the exposure-outcome relationship.

Our results revealed a higher prevalence of BP in LMICs when compared to higher income countries. This agrees with other reviews studies where the prevalence of LBP among studies from LMICs was higher than those from high income countries although the studies did not consider gender-specific effects [58, 59]. The higher odds of BP from pooled effect estimates from LMICs indicate that the impact of PDDL on women’s health may be more severe in LMICs. The biomechanical demands of domestic labor on women may be elevated where water infrastructure is lacking, or unreliable and water carrying/porterage is common [60]. Water carriage has long- and short-term impacts on musculoskeletal health of women [29].

In addition to water insecurity and carriage, daily domestic tasks such as caregiving activities and manual food processing could increase the risk of BP [27]. Women from developed economies may have less strenuous domestic labor than low-income women in LMICs as a result of differences in social/cultural expectations, better bargaining power due to higher paid income, increased use of mechanized household devices (e.g. dishwashers, laundry machines), and more social support/spousal involvement in performing domestic tasks. Similarly, middle-to-high income women in LMICs who have access to similar social and environmental resources might also face lower domestic labor health risks. These differences in domestic labor demands partially explain why domestic labor have traditionally been regarded as a ‘non-occupational or leisure-time’ physical activity with protective effects in published studies and reviews from developed countries [33–35].

### Methodological considerations: study inclusion and classification

The current study used well-defined inclusion and exclusion criteria to ensure that studies containing the intended target population (women) were selected. We excluded studies focused on women taking care of persons with disabilities and elderly women out of concern their level of exposure would not be generalizable to the overall population and because there is causal association between advanced age and low back pain [11]. We also excluded studies that did not fully provide exposure and outcome information to reduce the threat of differential misclassification of exposure or outcomes. We included studies examining acute and chronic back pain. We believe our inclusion/exclusion criteria were appropriate given that prior meta-analyses of non-occupational exposures and back pain have not assessed the impact of the domestic labor or focused on gendered effects.

### Strengths and limitations

The strengths of this review include: (1) extensive literature searches and inclusion of a study not published in English; (2) included studies were mostly of moderate to high quality and adjusted for relevant confounders; (3) identification of major sources of heterogeneity; (4) absence of publication bias; (5) Estimation of gender-specific association of domestic work with back pain. Almost all (six out eight) studies that sampled women engaged in paid work adjusted for physical demands of paid work and other relevant demographic factors during data analysis.

Several limitations should be considered when interpreting the findings from this study.

There are multiple factors that can confound the relationship between PDDL and BP. Although almost all included studies adjusted for relevant and common potential confounders (age, household size, paid work, income and number of children) of the association between PDDL and BP, it is possible that other potential confounders that were not assessed in included studies could distort the relationship between PDDL and BP. Assessing the impact of these unassessed potential confounders is important but beyond the capabilities of this meta-analysis.

Since the exposure and outcome information in most of the included studies were obtained using self-report methods, the results could be subject to reporting bias. Further, the temporal relationship between exposure to PDDL and BP cannot be established because most studies were cross-sectional, and we did not find any prospective cohort study to include in this meta-analysis [34]. Other potential sources of bias were those relating to study selection such as adequacy of sample size, response rate reporting, and comparability between respondents and non-respondents.

Furthermore, few of the included studies reported information regarding the validity of the instrument used ascertain PDDL. In addition, there were considerable methodological differences across the included studies in the measurement of domestic labor exposures, which may have led to misclassification of PDDL in our analyses [61]. Likewise, the definition, presenting symptoms, severity and period prevalence (ranges from one week to a year) of back pain differed across studies. These variations in exposure and outcome measurements across studies may have impacted both the magnitude and precision of our pooled OR estimates. Finally, since most studies ascertained both exposure and outcome by self-report, common method bias may have created the appearance of an association [62].

### Implications and recommendations for research

Results of the current study suggest an association between PDDL and BP, particularly among women in LMICs. Back pain is not typically a priority for mitigation because it is neither life-threatening nor as dangerous as other diseases prevalent in LMIC, yet it can cause long-lasting disability and declines in wellbeing and economic opportunity [58, 59]. Presently, gender-specific research investigating domestic exposures and musculoskeletal pain among women are limited [16, 22]. Presenting data on how domestic labor affects the musculoskeletal health of women will be important in designing future interventions (behavioral, infrastructural, and ergonomic) that can reduce burdens from domestic labor.

Most published large-scale studies that have assessed the relationship between domestic labor on musculoskeletal disorders mostly ascertain exposures using a woman’s role in the home (housewives) and self-report of exposure [33, 63, 64]. Relying solely on self-report information of exposures collected at one point in time from participants may be imprecise or lead to misclassification of exposure especially in routine daily activities such as domestic work [65]. Likewise, more information should be collected on pain experiences beside ‘absence or presence of pain’ to correctly ascertain the presence and severity pain in future studies [66, 67]. Future research should use objective measures of exposure, such as observation or instrument-based tools, to quantify PDDL so that exposure information is rigorous and can be standardized across studies [68]. Using a pain ratings scale, visual diagrams, specific recall period and probes to evaluate type of pain symptoms or level of impairment will be invaluable in reducing heterogeneity across studies, improving stability of estimates and generalization of findings. More longitudinal studies are needed to estimate the day-to-day variances in domestic labor, quantify the physical demands on women’s bodies, and understand the impact of unassessed potential confounders and moderators, before we can fully understand its causal relationship with BP.

Policymakers and labor organizations should put more concerted efforts in recognizing domestic labor as the occupational reality of many women, with health risks like those experienced in paid domestic and other artisan occupations. Adopting these approaches would significantly help in achieving the Sustainable Development Goals 5 of gender equity and women’s health.

## Conclusion

Results of the current study suggests that PDDL is associated with BP, especially among women from LMICs. Large prospective studies in LMICs are needed to critically examine or establish the relationship between domestic labor and BP.

## Supplementary Information

**Additional files 1: Table 1**. Multi-page table detailing the characteristics of Included Studies. **Table 2**. Risk of bias and overall quality rating using the new castle ottawa scale. **Table 3**. Quantifying heterogeneity between study sub-groups using Q-statistic. **Table 4**. List of excluded studies and reasons for exclusion. **Figure 1**. Baujat plot showing significant contribution to heterogeneity by Alzaharani 2019. **Figure 2**. Forest Plot with each study’s contribution to heterogeneity, omitted one at a time.  **Figure 3**. Forest plot showing sensitivity analysis of high-quality studies, investigating the association between physically demanding domestic labor and back pain in women. **Figure 4**. Funnel plot to Assess Publication Bias. **Appendix**.  Search terms culled from two databases.

## Data Availability

The data analysed were abstracted from published articles, other data generated are available in the supplemental file.

## Abbreviations

PDDL: Physically demanding domestic labor
BP: Back pain
LBP: Low back pain
OR: Odds ratio
CI: Confidence interval
LMICs: Low- and middle-income countries
MSDs: Musculoskeletal disorders
