# Supplementary material for: A meta-analysis of the association between physical demands of domestic labor and back pain among women
Source: BMC Womens Health. 2021 Apr 13;21:150. doi: 10.1186/s12905-021-01294-5 (PMC8045256; doi:10.1186/s12905-021-01294-5)
Supplement: Supplementary file 1 — Additional files 1: Table 1. Multi-page table detailing the characteristics of Included Studies. Table 2. Risk of bias and overall quality rating using the new castle ottawa scale. Table 3. Quantifying heterogeneity between study sub-groups using Q-statistic. Table 4. List of excluded studies and reasons for exclusion. Figure 1. Baujat plot showing significant contribution to heterogeneity by Alzaharani 2019. Figure 2. Forest Plot with each study’s contribution to heterogeneity, omitted one at a time. Figure 3. Forest plot showing sensitivity analysis of high-quality studies, investigating the association between physically demanding domestic labor and back pain in women. Figure 4. Funnel plot to Assess Publication Bias. Appendix. Search terms culled from two databases. [file 12905_2021_1294_MOESM1_ESM.docx]

| Additional File, Table 1. Characteristics of Included Studies | | | | | | | | | |
| --- | --- | --- | --- | --- | --- | --- | --- | --- | --- |
| Author, year, and (country of study) | Type of Study | Population | Demographics | % of women | Results stratified by gender | Type of Job Tasks specified | Confounders Adjusted | Description and measures Exposures | Description and Measures of outcome |
| Alzahrani 2019[1]  (England & Scotland) | Cross-sectional Study | Adults >/ 16 years. 32,087 women | Mean age=45.7years,  BMI, smoking status, education, ethnicity, employment status | Yes  50% | No | Tidying, cleaning, scrubbing and gardening | Age, Sex, smoking status, ethnicity, BMI, education, occupation, employment status, other physical activities (exercise) | OR, Low (<3.8) versus high (>=12.1) domestic activity (MET h/week). Low versus high/**Work intensity** | Chronic back pain |
| Ahlgren, 2012[2]  (Sweden) | Cross-sectional Study | Working women in blue-collar (253) and white-collar jobs (262) | Mean age=40.7 years (white collar), 44.3 years (blue-collar), education, family situation, employment status and working hours | Yes | Yes | Cleaning, carrying children, coking, washing clothes, regular shopping, washing dishes | Age, smoking, number of children, employment hours, blue collar work | OR, High versus low Engagement in domestic chores and caring for children/ **Work frequency** | Three months prevalence of Self-reported low back disorders |
| Fong, 2008[3] (Hong Kong) | Cross-sectional Study | 153 full time homemakers | Mean age=41.7 years, marital status, education, years in household work, number of children | Yes, 100 | NA | Carrying children, washing clothes, washing dishes, shopping | Demographics, other daily recreational activities | OR, Domestic activity (mean hours spent shopping& washing utensils)/ **Time** | 12-months prevalence of Self-perceived discomfort in the lower back |
| Geere, 2018[4]  (South Africa, Ghana and Vietnam) | Cross-sectional Survey | 789 women | Mean age= 29.7 years, type of water supply, general health functioning and disability | Yes  53.6 | No | Carrying water | Age and Sex | RR, History of water carriage (No history versus currently carries water)/**Biomechanical** | Upper back pain |
| Habib, 2012[5] (Lebanon) | Cross-sectional Study | 331 full time homemakers | Age, education, household income, size, number of children, smoking status | Yes, 100 | NA | Cleaning, carrying children, cooking, shopping | Age, education, paid work, income, household size | OR, working occasionally in Non-neutral versus Working all the time in Non-neutral postures (bending, kneeling and squatting) /**Biomechanical** | 12-months prevalence of low back pain |
| Habib, 2015[6] (Bangladesh) | Cross-sectional Study | 73 housewives | Age=20-45 years, education, | Yes, 100 | NA | Cooking, washing dishes, sweeping, collecting /carrying water. | None | OR, Trunk Flexion >45 degrees for more than 2 hours total per day/**Biomechanical** | 12-months prevalence of low back pain |
| Hubscher, 2014[7] (Australia) | Cross-sectional Study | 486 twins (277 females), ages 18-65 years. | Age, sex, smoking history | Yes, 57 | No | Vigorous gardening or heavy work around the yard | Age, Sex and smoking history | **OR, Total time** spent in domestic PA dichotomized as (more than 2 hours, less than 2 hours) | 1-month prevalence of self-reported Low Back pain |
| Josephson  2003[8] (Sweden) | Case-Control Study | 320 cases with low back pain  873 controls | Age, number of children, income, type of occupation, number of children | Yes, 100 | NA | Cleaning, carrying children, maintenance work, care of elderly parents | Psychosocial and physical factors of paid work, socio-demographic factors, and previous symptoms of LBP in the past three months | RR, Far too much and too little time on domestic work per day/**Time** | Clinically diagnosed low back pain |
| Rosano, 2004[9] (Italy) | Cross-sectional | 317 people, mean age=48.7 years, education, occupation | Sex, age, education | Yes, 96 | No | Making beds sewing cooking, cleaning, washing clothes/utensils, care giving, pet care | Age, education and occupation | OR, Self-reported frequency of domestic task per week/ **Frequency** | Self-reported low back pain |
| Malta  (2017)[10]  Brazil | Cross-sectional | 64,348 households | Age, education, race, residence, smoking, alcohol, BMI | No (can’t tell) | Yes | Heavy physical work at home, time spent on heavy physical activity at home | Age and education | OR, heavy physical domestic activity (Yes or No), /**work intensity** | Self-reported chronic back pain |
| Ranasinghe (2016)[11]  Sri Lanka | Cross-sectional | 1102 housewives | Age, marital status, number of children under-5, house ownership status, | Yes, 100 | NA | Cooking, handwash clothes, ironing, sweeping, shopping, childcare, cleaning | Child <5, married more than 10years, family income, self-rated health, psychosocial factors, long term illness | OR, working occasionally awkward versus working all the time in awkward postures/ **Biomechanical** | Self-reported low back pain |

**Additional File, Table 2.** Risk of Bias and Overall Quality Rating using the New Castle Ottawa Scale

| **Study** | **Study design** | **Selection** | **Comparability** | **Exposure/outcome** | | **Overall score** | **Quality** |
| --- | --- | --- | --- | --- | --- | --- | --- |
| Alzaharani 2019 | Cross-sectional | **** | ** | ** | 8 | | High |
| Geere 2018 | Cross-sectional | *** | ** | ** | 7 | | Moderate |
| Ahlgren 2012 | Cross-sectional | **** | ** | ** | 8 | | High |
| Hubscher 2014 | Cross-sectional | **** | ** | ** | 8 | | High |
| Habib 2015 | Cross-sectional | *** | None | ** | 5 | | Low |
| Habib 2012 | Cross sectional | **** | ** | ** | 8 | | High |
| Josephson 2003 | Case-Control | *** | ** | ** | 7 | | Moderate |
| Fong 2008 | Cross-sectional | ** | ** | ** | 6 | | Moderate |
| Rosano 2004 | Cross-sectional | **** | * | ** | 7 | | Moderate |
| Malta 2017 | Cross-sectional | **** | ** | ** | 8 | | High |
| Ranasinghe 2016 | Cross-sectional | **** | ** | ** | 8 | | High |

**Additional File, Table 3.**  Quantifying Heterogeneity Between Study Sub-Groups

| **Sub-Groups** | **Q-Statistic** | ***p*-value** |
| --- | --- | --- |
| Country Status | 11.16 | 0.0008 |
| Exposure Definition | 7.48 | 0.02 |
| Women-Specific Population | 0.54 | 0.46 |

**Additional File, Table 4.** Excluded Studies

| **Excluded Studies** | **Reason for Exclusion** |
| --- | --- |
| Apostoli et al. 2012[12]; Sanders and Morse, 2005[13]; Vincent and Hocking 2013[14] | Did not present means to calculate effect estimates |
| Backhausen et al. 2019[15]; Biglarian et al. 2012[16] | Only pregnant women |
| Mundt et al. 1993[17] | Included a sample of women comprising <50% of the total sample |
| Altinel et al. 2008[18]; Fazli et al. 2016[19]; Gupta and Nandini 2015[20]; Kalra and Bhatnagar 2017[21] | Defined exposures based on a women’s role but not the PDDW |
| Croft et al. 1999[22]; Takahashi et al. 2018[23]; Yip et al. 2004[24] | Focused on or included elderly women |
| Cavallari et al. 2016[25]; Cheung et al. 2006[26]; Krause et al. 2005[27] | Focused on paid housekeeping |
| Kopec et al. 2004[28]; Picavet and Schouten 2000[29] | Did not describe the types of domestic tasks |
| Cezar-Vaz et al. 2015[30]; Fernández et al. 2000[31] | Focused on other health outcomes |
| S. Mattioli et al. 2009[32]; Tang et al. 1999[33] | Focused on MSDs besides BP |

**Additional File, Figure 1.** Baujat Plot showing significant contribution to heterogeneity by Alzaharani 2019 (top-right corner)


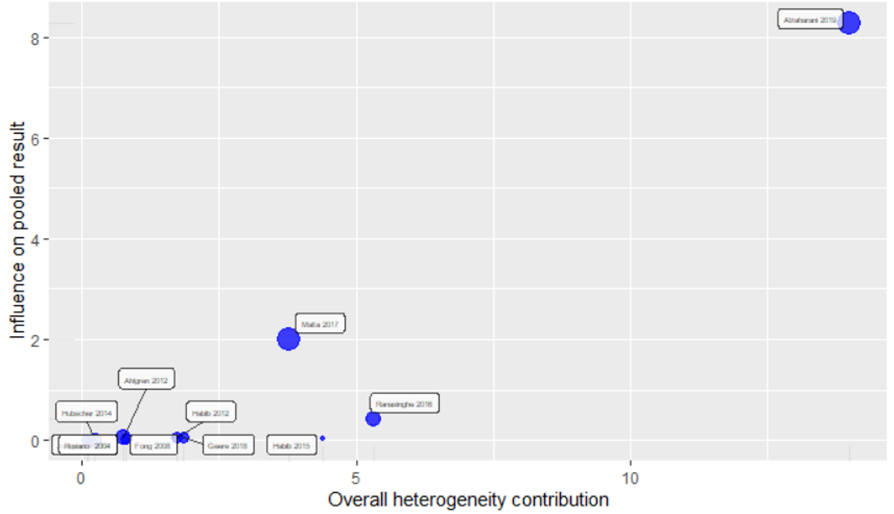


**Additional File, Figure 2.** Forest Plot with each study’s contribution to heterogeneity, omitted one at a time.


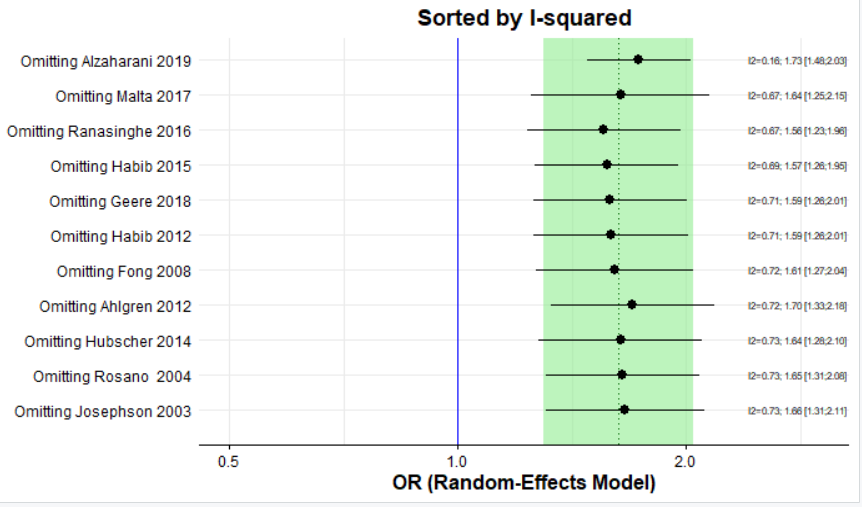


**Additional File, Figure 3.** Forest plot showing sensitivity analysis of high-quality studies, investigating the association between physically demanding domestic labor and back pain in women


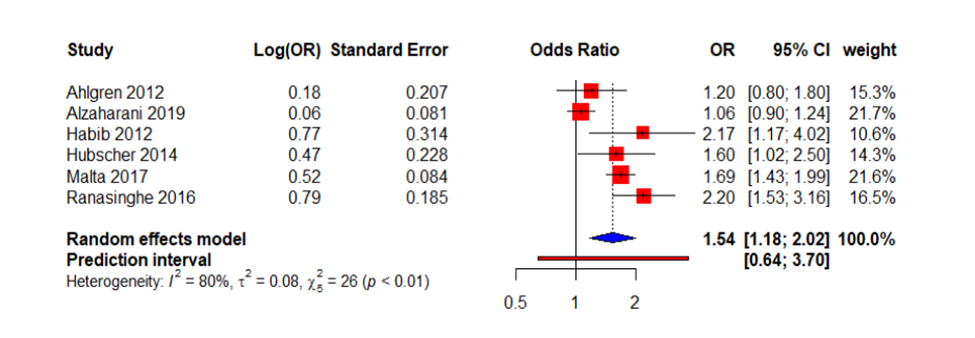


**Additional File, Figure 4.** Funnel plot


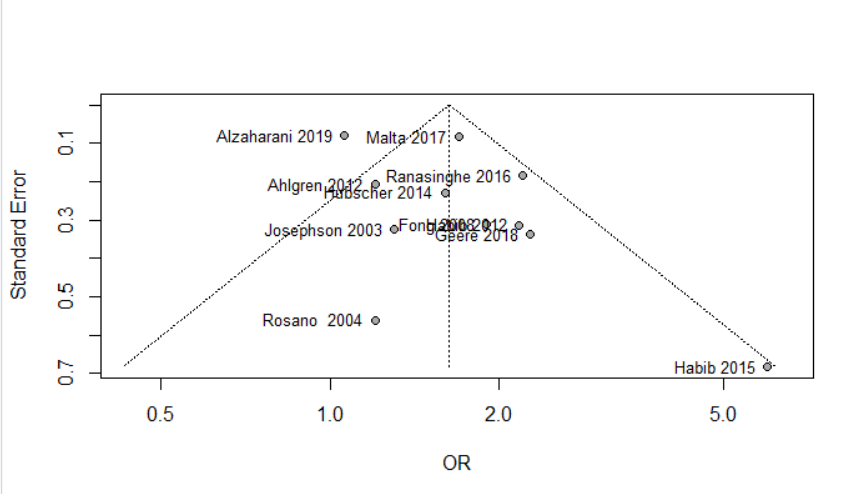


**Additional File, Appendix**

**PubMed**

**Population:** Housekeepers [All Fields] OR ("housekeeping"[MeSH Terms] OR "housekeeping"[All Fields] OR "housework"[All Fields]) OR homecare [All Fields] OR "domestic"[All Fields]) OR housewives [All Fields]

**Outcome:**(Musculoskeletal[All Fields] AND ("injuries"[Subheading] OR "injuries"[All Fields] OR "wounds and injuries"[MeSH Terms] OR ("wounds"[All Fields] AND "injuries"[All Fields]) OR "wounds and injuries"[All Fields])) OR ("musculoskeletal diseases"[MeSH Terms] OR ("musculoskeletal"[All Fields] AND "diseases"[All Fields]) OR "musculoskeletal diseases"[All Fields] OR ("musculoskeletal"[All Fields] AND "disorders"[All Fields]) OR "musculoskeletal disorders"[All Fields]) OR ("occupational injuries"[MeSH Terms] OR ("occupational"[All Fields] AND "injuries"[All Fields]) OR "occupational injuries"[All Fields]) OR (("upper extremity"[MeSH Terms] OR ("upper"[All Fields] AND "extremity"[All Fields]) OR "upper extremity"[All Fields]) AND ("injuries"[Subheading] OR "injuries"[All Fields] OR (("lower extremity"[MeSH Terms] OR ("lower"[All Fields] AND "extremity"[All Fields]) OR "lower extremity"[All Fields]) AND ("injuries"[Subheading] OR "injuries"[All Fields] OR "pain"[All Fields]) OR ("back pain"[MeSH Terms] OR ("low"[All Fields] AND "back"[All Fields] AND "pain"[All Fields]) OR "low back pain"[All Fields])

**Exposure:**(("ergonomics"[MeSH Terms] OR "ergonomics"[All Fields] OR "ergonomic"[All Fields]) AND ("risk"[MeSH Terms] OR "risk"[All Fields])) OR (("physical examination"[MeSH Terms] OR ("physical"[All Fields] AND "examination"[All Fields]) OR "physical examination"[All Fields] OR "physical"[All Fields]) AND load[All Fields]) OR ("lifting"[MeSH Terms] OR "lifting"[All Fields]) OR ("lifting"[MeSH Terms] OR "lifting"[All Fields] OR "carrying"[All Fields]) OR (("manuals as topic"[MeSH Terms] OR ("manuals"[All Fields] AND "topic"[All Fields]) OR "manuals as topic"[All Fields] OR "manual"[All Fields]) AND task[All Fields]) OR (("physical examination"[MeSH Terms] OR ("physical"[All Fields] AND "examination"[All Fields]) OR "physical examination"[All Fields] OR "physical"[All Fields]) AND ("risk"[MeSH Terms] OR "risk"[All Fields])) OR ("posture"[MeSH Terms] OR "posture"[All Fields])

**Embase:**

('housewife'/exp OR housewife OR 'housekeeping'/exp OR housekeeping OR 'cleaning'/exp OR cleaning OR 'household'/exp OR household OR 'home care'/exp OR 'home care' OR 'caregiver burden'/exp OR 'caregiver burden') AND ('musculoskeletal disease'/exp OR 'musculoskeletal disease' OR 'musculoskeletal pain'/exp OR 'musculoskeletal pain' OR 'injury'/exp OR injury OR 'occupational disease'/exp OR 'occupational disease' OR 'ergonomics'/exp OR ergonomics OR 'physical load'/exp OR 'physical load' OR (physical AND ('load'/exp OR load)) OR 'weight bearing'/exp OR 'weight bearing' OR 'manual labor'/exp OR 'manual labor' OR 'biomechanics'/exp OR biomechanics OR 'load carrying capacity'/exp OR 'load carrying capacity') AND 'human'/de AND ('cohort analysis'/de OR 'cross-sectional study'/de OR 'longitudinal study'/de OR 'prospective study'/de OR 'questionnaire

**REFERENCES**

1. Alzahrani H, Shirley D, Cheng SWM, Mackey M, Stamatakis E: **Physical activity and chronic back conditions: A population-based pooled study of 60,134 adults**. *Journal of Sport and Health Science* 2019.

2. Ahlgren C, Malmgren Olsson EB, Brulin C: **Gender analysis of musculoskeletal disorders and emotional exhaustion: interactive effects from physical and psychosocial work exposures and engagement in domestic work**. *Ergonomics* 2012, **55**(2):212-228.

3. Fong KN, Law CY: **Self-perceived musculoskeletal complaints: relationship to time use in women homemakers in Hong Kong**. *J Occup Rehabil* 2008, **18**(3):273-281.

4. Geere JA, Bartram J, Bates L, Danquah L, Evans B, Fisher MB, Groce N, Majuru B, Mokoena MM, Mukhola MS *et al*: **Carrying water may be a major contributor to disability from musculoskeletal disorders in low income countries: a cross-sectional survey in South Africa, Ghana and Vietnam**. *Journal of global health* 2018, **8**(1):010406.

5. Habib RR, El Zein K, Hojeij S: **Hard work at home: musculoskeletal pain among female homemakers**. *Ergonomics* 2012, **55**(2):201-211.

6. Habib MM, Rahman SU: **Musculoskeletal symptoms (MSS) and their associations with ergonomic physical risk factors of the women engaging in regular rural household activities: A picture from a rural village in Bangladesh**. *Work (Reading, Mass)* 2015, **50**(3):347-356.

7. Hubscher M, Ferreira ML, Junqueira DR, Refshauge KM, Maher CG, Hopper JL, Ferreira PH: **Heavy domestic, but not recreational, physical activity is associated with low back pain: Australian Twin low BACK pain (AUTBACK) study**. *Eur Spine J* 2014, **23**(10):2083-2089.

8. Josephson M, Ahlberg G, Harenstam A, Svensson H, Theorell T, Wiktorin C, Vingard E: **Paid and unpaid work, and its relation to low back and neck/shoulder disorders among women**. *Women & health* 2003, **37**(2):17-30.

9. Rosano A, Moccaldi R, Cioppa M, Lanzieri G, Persechino B, Spagnolo A: **[Musculoskeletal disorders and housework in Italy]**. *Annali di igiene : medicina preventiva e di comunita* 2004, **16**(3):497-507.

10. Malta DC, Oliveira MM, Andrade S, Caiaffa WT, Souza MFM, Bernal RTI: **Factors associated with chronic back pain in adults in Brazil**. *Rev Saude Publica* 2017, **51**(suppl 1):9s.

11. Ranasinghe PD, Atukorala I, Samaranayake A, Gunawardana NS: **SAT0504 Prevalence and Correlates of Household Work Related Musculoskeletal Disorders in Low Back Region among Sri Lankan Housewives**. *Annals of the Rheumatic Diseases* 2016, **75**(Suppl 2):852-852.

12. Apostoli P, Sala E, Curti S, Cooke RM, Violante FS, Mattioli S: **Loads of housework? Biomechanical assessments of the upper limbs in women performing common household tasks**. *Int Arch Occup Environ Health* 2012, **85**(4):421-425.

13. Sanders MJ, Morse T: **The Ergonomics of Caring for Children: An Exploratory Study**. *American Journal of Occupational Therapy* 2005, **59**(3):285-295.

14. Vincent R, Hocking C: **Factors that might give rise to musculoskeletal disorders when mothers lift children in the home**. *Physiother Res Int* 2013, **18**(2):81-90.

15. Backhausen MG, Bendix JM, Damm P, Tabor A, Hegaard HK: **Low back pain intensity among childbearing women and associated predictors. A cohort study**. *Women Birth* 2019, **32**(4):e467-e476.

16. Biglarian A, Seifi B, Bakhshi E, Mohammad K, Rahgozar M, Karimlou M, Serahati S: **Low back pain prevalence and associated factors in Iranian population: findings from the national health survey**. *Pain Res Treat* 2012, **2012**:653060.

17. Mundt DJ, Kelsey JL, Golden AL, Pastides H, Berg AT, Sklar J, Hosea T, Panjabi MM: **An epidemiologic study of non-occupational lifting as a risk factor for herniated lumbar intervertebral disc. The Northeast Collaborative Group on Low Back Pain**. *Spine* 1993, **18**(5):595-602.

18. Altinel L, Kose KC, Ergan V, Isik C, Aksoy Y, Ozdemir A, Toprak D, Dogan N: **[The prevalence of low back pain and risk factors among adult population in Afyon region, Turkey]**. *Acta orthopaedica et traumatologica turcica* 2008, **42**(5):328-333.

19. Fazli B, Ansari H, Noorani M, Jafari SM, Sharifpoor Z, Ansari S: **The Prevalence of Musculoskeletal Disorders and its Predictors among Iranians’ Housewives**. *International Journal of Epidemiologic Research* 2016, **3**(1):53-62.

20. Gupta G, Nandini N: **Prevalence of low back pain in non working rural housewives of Kanpur, India**. *International journal of occupational medicine and environmental health* 2015, **28**(2):313-320.

21. Kalra S, Bhatnagar B: **Prevalence of Musculoskeletal Disorder among Housewives**. In*: 2017*; 2017.

22. Croft PR, Papageorgiou AC, Thomas E, Macfarlane GJ, Silman AJ: **Short-term physical risk factors for new episodes of low back pain. Prospective evidence from the South Manchester Back Pain Study**. *Spine* 1999, **24**(15):1556-1561.

23. Takahashi A, Kitamura K, Watanabe Y, Kobayashi R, Saito T, Takachi R, Kabasawa K, Oshiki R, Tsugane S, Iki M *et al*: **Epidemiological profiles of chronic low back and knee pain in middle-aged and elderly japanese from the Murakami cohort**. *Journal of Pain Research* 2018, **11**:3161-3169.

24. Yip YB, Ho SC, Chan SG: **Identifying risk factors for low back pain (LBP) in Chinese middle-aged women: a case-control study**. *Health care for women international* 2004, **25**(4):358-369.

25. Cavallari JM, Ahuja M, Dugan AG, Meyer JD, Simcox N, Wakai S, Garza JL: **Differences in the prevalence of musculoskeletal symptoms among female and male custodians**. *American journal of industrial medicine* 2016, **59**(10):841-852.

26. Cheung K, Gillen M, Faucett J, Krause N: **The prevalence of and risk factors for back pain among home care nursing personnel in Hong Kong**. *American Journal of Industrial Medicine* 2006, **49**(1):14-22.

27. Krause N, Scherzer T, Rugulies R: **Physical workload, work intensification, and prevalence of pain in low wage workers: Results from a participatory research project with hotel room cleaners in Las Vegas**. *American Journal of Industrial Medicine* 2005, **48**(5):326-337.

28. Kopec JA, Sayre EC, Esdaile JM: **Predictors of Back Pain in a General Population Cohort**. *Spine* 2004, **29**(1):70-77.

29. Picavet HS, Schouten JS: **Physical load in daily life and low back problems in the general population-The MORGEN study**. *Preventive medicine* 2000, **31**(5):506-512.

30. Cezar-Vaz MR, Bonow CA, da Silva MR: **Mental and Physical Symptoms of Female Rural Workers: Relation between Household and Rural Work**. *International journal of environmental research and public health* 2015, **12**(9):11037-11049.

31. Fernández E, Schiaffino A, Martí M: **Influencia del trabajo doméstico sobre la salud y la utilización de servicios sanitarios en mujeres con trabajo remunerado y amas de casa**. *Gaceta Sanitaria* 2000, **14**(4):287-290.

32. Mattioli S, Baldasseroni A, Bovenzi M, Curti S, Cooke RMT, Campo G, Barbieri PG, Ghersi R, Broccoli M, Cancellieri MP *et al*: **Risk factors for operated carpal tunnel syndrome: a multicenter population-based case-control study**. *BMC public health* 2009, **9**:343-343.

33. Tang X, Zhuang L, Lu Z: **Carpal tunnel syndrome: a retrospective analysis of 262 cases and a one to one matched case-control study of 61 women pairs in relationship between manual housework and carpal tunnel syndrome**. *Chinese medical journal* 1999, **112**(1):44-48.
